# Intradiploic Hematoma in a Hemophilic Patient: Hemophilic Pseudotumor of Calvarium

**DOI:** 10.4274/tjh.2016.0254

**Published:** 2017-03-01

**Authors:** Hakan Hanımoğlu, Zafer Başlar

**Affiliations:** 1 İstanbul Bilim University Faculty of Medicine, Department of Neurosurgery, İstanbul, Turkey; 2 İstanbul University Cerrahpaşa Faculty of Medicine, Department of Internal Medicine, Division of Hematology, İstanbul, Turkey

**Keywords:** Hemophilia A, Intradiploic hematoma, Coagulopathy, Intraosseous

## TO THE EDITOR,

Pseudotumors are results of repeated hemorrhage into soft tissues, the subperiosteum, or a site of bone fracture with inadequate resorption of the extravasated blood. We describe a patient with a huge hemophilic pseudotumor of calvarium, which occurs very rarely.

A 14-year-old boy with mild hemophilia A (FVIII coagulant activity: 5.8%) without inhibitor presented with epileptic seizure 7 years ago. The patient was known to be hemophiliac from birth after a birth injury and brain damage had occurred. He was mentally retarded and had habitual head-hitting behavior. His family noticed progressively enlarging painless scalp swelling on his head.

**Figure 1:**
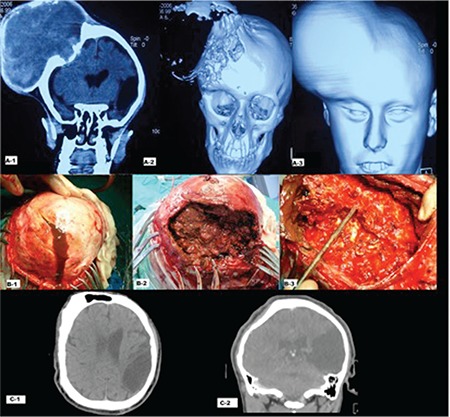
A-1, A-2, A-3: Multidetector computed tomography scan with reconstruction shows large lytic intradiploic lesion with expansion and scalloping of the bony margins; please note that the inner and outer tables are separated and destructed. B-1, B-2, B-3: Intraoperative images; evacuated lesion was mud-like, inner table was protected. C1, C2: Sagittal and coronal computed tomography images after 7 years; good and acceptable remodeling of the calvarium is seen.

There was obvious asymmetry of the head and face (Figure 1, A-3). His neurological examination was normal and radiological investigations did not reveal any other pathology. A computed tomography (CT) scan showed a large lesion with a mass effect over the underlying brain (Figure 1, A-1 and A-2).

Surgery was carried out with coagulation factor replacement (FVIII). During surgery a skin flap was done and the thinned outer table was incised (Figure 1, B-1). Mud-like material and a liquefied clot were evacuated (Figure 1, B-2). The thin and elastic inner wall was not removed to avoid postoperative complications (Figure 1, B-3).

Following surgery, antiepileptic medication was continued and short-term prophylaxis (30 IU/kg, three times a week) was applied for 8 weeks. At the 7-year follow-up of the patient, he was free of seizures and a CT scan of the patient showed that acceptable calvarial remodeling had occurred (Figure 1, C-1 and C-2).

Proximal pseudotumors may destroy the soft tissues, erode the bone, and cause serious vascular and/or nerve damage [1]. Reduction of the pseudotumor and chronic joint disease is achieved by prophylactic treatment in severe hemophilia.

Calvarial localization of a pseudotumor is unusual [2]. Inflammation due to hematoma causes immune reaction and affects nearby tissues. The skull tables provide natural protection from soft tissues being eroded [3]. All intradiploic lesions should be suspected to be hematomas unless proven otherwise in patients with coagulopathies [2].

Total surgical removal of the hematoma is the treatment of choice. Some authors recommend cosmetic cranioplasty within the same surgical procedure [4]. However, most of them prefer to preserve the intact inner table [5]. According to us, the elastic inner table must be preserved to avoid postoperative complications. Acceptable bone remodeling was seen in the seventh year of follow-up in control CT images. However, a noncompressible inner table must be excised.

In summary, intradiploic hematoma must be expected when an intradiploic lesion is seen with hemophilia. The main part of the surgery is the preservation of the inner table of the cranium in hemophilic patients. Bone remodeling gives good results with time.
